# Understanding the basis of major depressive disorder in oncological patients: Biological links, clinical management, challenges, and lifestyle medicine

**DOI:** 10.3389/fonc.2022.956923

**Published:** 2022-09-16

**Authors:** Oscar Fraile-Martinez, Miguel A. Alvarez-Mon, Cielo Garcia-Montero, Leonel Pekarek, Luis G. Guijarro, Guillermo Lahera, Miguel A. Saez, Jorge Monserrat, Domitila Motogo, Javier Quintero, Melchor Alvarez-Mon, Miguel A. Ortega

**Affiliations:** ^1^ Department of Medicine and Medical Specialities, Faculty of Medicine and Health Sciences, University of Alcalá, Alcala de Henares, Spain; ^2^ Ramón y Cajal Institute of Sanitary Research (IRYCIS), Madrid, Spain; ^3^ Department of Psychiatry and Mental Health, Hospital Universitario Infanta Leonor, Madrid, Spain; ^4^ Oncology Service, Guadalajara University Hospital, Guadalajara, Spain; ^5^ Unit of Biochemistry and Molecular Biology, Department of System Biology, Centro de Investigación Biomédica en Red en el Área temática de Enfermedades Hepáticas (CIBEREHD), University of Alcalá, Alcala de Henares, Spain; ^6^ Psychiatry Service, Center for Biomedical Research in the Mental Health Network, University Hospital Príncipe de Asturias Centro de Investigación Biomédica en Red en el Área temática de Salud Mental (CIBERSAM), Alcalá de Henares, Spain; ^7^ Pathological Anatomy Service, Central University Hospital of Defence-UAH Madrid, Alcala de Henares, Spain; ^8^ Department of Legal Medicine and Psychiatry, Complutense University, Madrid, Spain; ^9^ Immune System Diseases-Rheumatology, Oncology Service an Internal Medicine, Centro de Investigación Biomédica en Red en el Área temática de Enfermedades Hepáticas (CIBEREHD), University Hospital Príncipe de Asturias, Alcala de Henares, Spain; ^10^ Cancer Registry and Pathology Department, Principe de Asturias University Hospital, Alcala de Henares, Spain

**Keywords:** cancer, depression, lifestyle medicine, multidisciplinary approaches, clinical challenges, Translational medicine

## Abstract

In recent years, the incidence of different types of cancer and patient survival have been rising, as well as their prevalence. The increase in survival in recent years exposes the patients to a set of stressful factors such as more rigorous follow-up and more aggressive therapeutic regimens that, added to the diagnosis of the disease itself, cause an increase in the incidence of depressive disorders. These alterations have important consequences for the patients, reducing their average survival and quality of life, and for these reasons, special emphasis has been placed on developing numerous screening tests and early recognition of depressive symptoms. Despite that cancer and major depressive disorder are complex and heterogeneous entities, they also share many critical pathophysiological mechanisms, aiding to explain this complex relationship from a biological perspective. Moreover, a growing body of evidence is supporting the relevant role of lifestyle habits in the prevention and management of both depression and cancer. Therefore, the present study aims to perform a thorough review of the intricate relationship between depression and cancer, with a special focus on its biological links, clinical management, challenges, and the central role of lifestyle medicine as adjunctive and preventive approaches to improve the quality of life of these patients.

## Introduction

Major depressive disorder (MDD) is one of the most important and disabling psychiatric illnesses affecting a large proportion of today’s population. Indeed, it is estimated that approximately more than 260 million people in the world suffer from different degrees of MDD (1). Throughout history, both the diagnosis and treatment of MDD have changed over time. The causes that explain its origin are multifactorial, but a relationship between MDD and cancer has been described for years, both before and after diagnosis, which has led basic and clinical researchers to describe the underlying pathophysiology. In recent years, the incidence of patients affected by some type of cancer has increased gradually, estimating that by 2030, 26 million malignant neoplasms and up to 17 million deaths will be reported worldwide (2). The increase in incidence is in turn accompanied by an increase in prevalence due to both early diagnosis and improved treatment, which have improved the prognosis after diagnosis. The combination of the use of immunotherapy with chemoradiotherapy has made it possible to optimally address malignant tumors that previously had a dire prognosis (3). We cannot forget that both the diagnosis and the disease itself carry a great emotional burden that causes many of the patients to present different psychiatric pathologies such as depression, anxiety, or post-traumatic stress disorder during or after having overcome a malignant neoplasm (4). Numerous studies have shown how depression after cancer diagnosis causes a delay in the start of treatment, a decrease in quality of life, and an increase in the number of suicide attempts (5). It should be noticed that compared to the general population, the overall risk of cancer patients being diagnosed with MDD throughout the course of the disease is two to four times more frequent (6), although the available literature that assesses the prevalence of depression in cancer patients varies from 5% to 60% according to different authors (7).

A growing body of evidence has found common biological mechanisms between cancer and depression. In the same way, an accurate diagnosis and adequate clinical management of oncological patients with MDD are critical, as well as different approaches to prevent their progression. In this sense, lifestyle medicine represents a very important approach with potential benefits in both alleviating and preventing the development of depressive symptoms in cancer patients. Therefore, the purpose of this study is to delve into the different mechanisms that relate depression and cancer, as well as their translation in the clinic, including the challenges to overcome in this field and giving a central role to interventions in the lifestyle to prevent and achieve a better quality of life and clinical management of these patients.

## Establishing the biological links between cancer and major depressive disorder

Firstly, the biological basis of both cancer and MDD should be completely unraveled in order to identify and describe their biological links and potential preventive/therapeutic approaches. The etiopathogenesis and pathophysiological mechanisms of cancer and MDD are quite heterogeneous and intricate. Cancer is a complex entity, with unique clinical manifestations characterized by several molecular and cellular alterations brilliantly described by Hanahan and Weinberg (8). Some of the most important features of cancer include sustained proliferation, genome instability, tumor-promoting inflammation, immune evasion, apoptosis avoidance, altered metabolism, and metastasis. Moreover, the proper initiation and progression of tumoral cells entail not only local but also global consequences, affecting the different organs and systems of the organism (9, 10). Following the International Agency for Research on Cancer (IARC), some types of cancer may appear due to some viral, bacterial, parasitic, or fungal infections (11). The heritability of cancer in twins has been estimated at 30%, although the percentage differs according to the type of cancer (12). Other important contributors to the etiology of cancer are lifestyle factors like smoking, alcohol consumption, diet, obesity, and sedentarism, together with hormonal and reproductive factors, pharmaceuticals, or excessive/low sunlight exposure (13).

In the case of MDD, it is a multifactorial disorder frequently manifested as a depressed mood or loss of interest or pleasure (anhedonia) accompanied by a set of somatic and vegetative items like feelings of worthlessness, lack of energy, poor concentration, appetite changes, sleep disturbances, or suicidal thoughts (14). Patients with MDD often exhibit several functional and structural changes in different regions of the brain and neuronal networks, as well as the altered immune system and gut microbiota and multiple systemic alterations (15). MDD onset is associated with many genetic and environmental factors. In a similar manner to cancer, cumulative evidence has estimated that the heritability of MDD is about 30% to 40% (16). Moreover, unhealthy lifestyle factors like low physical activity levels, improper dietary habits, or sleep disturbances are also related to the onset of MDD (17). However, different types of psychological stress, including early life stress (ELS) or chronic stress, are thought to be the major contributors to MDD onset (18). Although there are unique and different pathological signatures in cancer and MDD, being diagnosed with cancer is commonly related to suffering from this psychiatric condition, and in turn, suffering from MDD appears to significantly increase the risk of cancer incidence as well as poorer cancer survival, and higher cancer-specific mortality (19). Moreover, not only psychological stress but also other mechanisms have been described in the connection between cancer and MDD, including circadian disruption, inflammation, gut dysbiosis, and abnormal neurotransmission (20). Indeed, there is an intricate integration of signals between the nervous, immune, and endocrine systems along with the gut microbiota and the psychological functioning of the individual. This is collected under the term psychoneuroimmunoendocrinology (PNIE), resulting in the pivotal etiopathogenic link between complex diseases like cancer and psychiatric disorders like MDD (21). Thus, despite that we will summarize the biological mechanisms in 1) psychological stress and circadian disruption, 2) inflammation and gut dysbiosis, and 3) abnormal neurotransmission, it is important to understand that all these factors are not influencing alone but coordinately in the relationship between depression and cancer.

### Psychological stress and circadian disruption

As above mentioned, being diagnosed with cancer is a strong source of psychological stress for the patients, which can be an important driver of MDD for oncological patients, especially for those with higher stress susceptibility or chronic stress prior to diagnosis. Psychological stress induces the hyperactivation of the hypothalamic–pituitary–adrenal (HPA) axis, leading to an increase of corticotropin-releasing factor (CRF), adrenocorticotropic hormone (ACTH), and cortisol. Importantly, these components are associated with a set of structural and functional changes in the brain and the entire organism (22, 23). Notwithstanding that the IARC does not recognize psychological stress as a cause of any type of cancer, compelling evidence is starting to suggest that, at least, psychological stress and carcinogenesis are tightly associated (24). Nowadays, it is widely accepted that the hyperactivation of the HPA axis and the sympathetic nervous system (SNS) drives decline and dysfunction in the hippocampus and prefrontal cortex along with hyperactivation of the amygdala, which is associated with MDD development (25). Moreover, previous studies have noticed that HPA dysregulation is responsible for the concurrence of several symptoms in patients with advanced stages of lung cancer (26). Li et al. (27) studied a set of genes involved in the alteration of the HPA axis in patients with breast cancer in order to identify women at risk of suffering from psychiatric and depressive symptoms. Interestingly, they found specific polymorphic variants in some critical genes like FKBP5 (rs9394309), NR3C2 (rs5525), and CRHR1 (rs12944712), which can be used to explain the relevance of the HPA axis in women with breast cancer. Systemically, the hyperactivation of the HPA axis is tightly related to inflammation and gut dysbiosis as it will be later discussed. Likewise, an altered HPA axis is frequently related to abnormal biorhythms of cortisol release due to an impaired action of the different circadian clocks (28). Regarding cortisol, the levels of this hormone are high in the morning and diminish in the evening/night. However, chronic stress mismatches the circadian oscillation of cortisol levels, something that could be observed in patients with MDD (20). In turn, the relevance of chronobiological variations in cortisol levels has been demonstrated in different neoplasms and how can they influence the progression of these tumors (29, 30). What is more, patients with cancer exhibit higher plasma cortisol concentrations at 8 AM and 8 PM, and the relative diurnal variation of cortisol was found to be decreased in cancer patients with depression, indicating a disturbed circadian function of the HPA axis, with a sensitivity of 81% and specificity of 88% at a cutoff value of 33.5% (31). Moreover, patients with cancer may present multiple alterations affecting critical genes involved in circadian regulation, especially at advanced stages (32, 33). In this sense, melatonin is a master regulator of circadian rhythms, especially regulating the light–dark cycles in the suprachiasmatic nuclei (SCNs) of the hypothalamus (34). This hormone is crucial for several physiological processes in the brain and the entire organism, and its dysregulation appears to be involved in the onset and development of both MDD and cancer (35, 36). Zaki et al. (37) observed that serum levels of melatonin in 45 women with breast cancer patients correlated significantly with self-reported sleep quality and psychometric profiles of depression. Because of that, there have been some studies showing the promising role of melatonin in reducing the risk of depression in patients with cancer (38). However, to fully understand the effects of chronic stress, HPA dysfunction, and circadian alterations in patients with cancer and MDD, the role of the immune system and gut microbiota as pivotal modulators of several processes needs to be explored.

### Inflammation and gut dysbiosis

The immune system and gut microbiota are two major components involved in several physiological processes, being both strongly interconnected (39). Patients with cancer often exhibit an important systemic dysregulation of immune cells and cytokines (40), as well as an abnormal gut microbiota composition (dysbiosis), which in turn has detrimental translational consequences (41). This inflammation can also affect the brain (neuroinflammation), which may represent an adaptive response of the brain to peripheral challenges and tumor growth (42). An exacerbated inflammation is a major feature of MDD, especially in the brain (neuroinflammation), which is associated with different pathophysiological mechanisms implicated in the etiopathogenesis of depression (43). Moreover, patients with MDD present an increase in systemic markers of inflammation, frequently accompanied by gut dysbiosis and an enhanced bacterial translocation (44–46). Likewise, chronic stress has important effects on the immune system and gut microbiota, leading to persistent chronic inflammation and gut dysbiosis, which may be associated with cancer development and progression as well (47).

The gut microbiota affects the brain through several mechanisms through the widely known microbiota–gut–brain (MGB) axis. This axis comprises neurotransmitter/neuropeptide regulation, immunomodulatory effects, and the production of unique microbial metabolites like short-chain fatty acids (SCFAs), thereby regulating the brain in physiological and pathological conditions (48, 49). Interestingly, the immune system, gut microbiota, and their metabolites are essential drivers of tumorigenesis and cancer progression through several mechanisms (41). Gonzalez-Mercado et al. (50) studied the correlation between different bacterial taxa with the onset of depressive symptoms in patients with colorectal cancer after submitting to chemotherapy and radiotherapy. They observed that the relative abundance of *Gemella*, Bacillales Family XI, *Actinomyces*, *Streptococcus*, *Lactococcus*, *Weissella*, and Leuconostocaceae were positively correlated with depressive symptoms, whereas *Coprobacter*, *Intestinibacter*, *Intestinimonas*, Lachnospiraceae, *Phascolarctobacterium*, *Ruminiclostridium*, Ruminococcaceae, *Tyzzerella*, and *Parasutterella* were negatively correlated. In accordance with these results, prior studies have demonstrated that depressed patients with breast cancer exhibit lower gut microbiota diversity, with an augmented relative abundance of Proteobacteria and *Escherichia–Shigella* than non-depressed patients, with a poor-quality diet essential to understanding these changes (51). Likewise, patients with gastrointestinal cancer and depressive symptoms showed a reduced relative abundance of *Gemmiger*, *Ruminococcus*, and *Veillonella* and a lower alpha diversity in fecal samples (52). Notwithstanding that it is difficult to find out if gut microbiota is a cause or a consequence of the link between cancer and MDD, it seems that oncological patients with MDD may present a distinctive gut microbiota signature in comparison to those without depression. Further studies are needed to unravel biological mechanisms and applications to different types of tumors, as it may represent a promising translational approach for cancer and depressive symptoms (53). In this line, we propose to conduct further studies in the field of microbial metabolites like the abovementioned SCFAs, as these represent a potential opportunity to address not only the MGB axis but also some types of tumors like colorectal cancer (54).

The immune system may also play a key role in the link between MDD and cancer. For instance, there are plenty of proinflammatory cytokines produced peripherally by macrophages and lymphocytes, and centrally by astrocytes and microglia, facilitating the onset of depressive symptoms. In this sense, it is known that excessive proinflammatory cytokine production is related to abnormal monoamine neurotransmitter metabolism, limbic system activity, and HPA dysregulation (55). In accordance with this statement, it is known that immune system–tumor interaction causes the release of proinflammatory cytokines such as IL-6 and IL-18, which numerous studies have shown to be related to depressive symptoms (56, 57). For example, authors such as Jehn et al. have described how IL-6 can cause through the activation of the HPA axis (58). In fact, previous studies have found that depressed patients with cancer display enhanced serum levels of IL-6, which is considered a major biomarker of MDD, with a sensitivity of 79% and a specificity of 87% (31). The presence of other cytokines such as IL-1β in the hypothalamus appears to be related to the cachexia–anorexia syndrome that accompanies numerous tumors (59, 60). TNF-α, C-reactive protein (CRP), and serum soluble interleukin-2 receptor (sIL2r) have also been associated with MDD in patients with cancer, directly modulating the HPA axis (61). Likewise, previous studies have described how there are neuroplastic modifications related to the production of different chemotactic cytokines such as CXC or CX3C that cause alterations in neuronal processes, neuronal plasticity, neurogenesis, and synaptic transmission, which can induce the development of depression (62). Moreover, animal models have demonstrated the relevance of social isolation in the sensitization to the detrimental effects of tumor-derived inflammation and proinflammatory cytokines, which may explain the onset of depressive symptoms like anhedonia in oncological patients (63). In turn, the presence of depression and the abovementioned chronic stress is associated with decreased cytotoxic T-cell and natural killer cell activities, thereby affecting immune surveillance of tumors and favoring the development and accumulation of somatic mutations and genomic instability (64). Thus, tumoral cells appear to present a more aggressive phenotype when it co-occurs with MDD and is thus associated with a poorer prognosis (61). Likewise, sustained inflammation, gut dysbiosis, and chronic stress may lead to a wide variety of systemic endocrine changes, including insulin resistance, sex hormone dysregulation, or an aberrant functioning of the renin–angiotensin–aldosterone system (RAAS) (65). Indeed, these changes represent a critical link between cancer and MDD, particularly in the context of an aberrant immune system and gut dysbiosis. Moreover, cancer therapy may be associated with multiple endocrine changes that in turn may lead to the appearance of MDD (66).

Overall, the effects of gut dysbiosis and immune dysfunction in patients with cancer and MDD are numerous, and there is a tight interplay between these components, along with chronic stress.

### Abnormal neurotransmission

The third mechanism involved in the relationship between cancer and MDD is an abnormal neurotransmitter and neuropeptide metabolism/action. In this sense, we must highlight the role of monoamines as pivotal neurotransmitters dysregulated in patients with cancer.

Monoamines, represented by dopamine, norepinephrine, and serotonin (5-HT), are central neurotransmitters that play a central role in the pathophysiology of MDD (67). Despite traditionally that it was considered the major cause of depression—mainly because many antidepressants target monoamines such as the widely known serotonin selective reuptake inhibitors (SSRIs)—nowadays, the role of monoamines in the onset of MDD has been taken more cautiously (68). It is well-known that these neurotransmitters mediate many cognitive and emotional domains, including mood, thoughts, attention, behavior, and sensory experiences (69). Thus, their altered levels of MDD are associated with multiple symptoms. For instance, low levels of 5-HT are linked to behavioral changes and somatic dysfunctions (including depressed mood, appetite, sleep, sex, pain response, body temperature, and circadian rhythm); abnormal dopamine action can lead to anhedonia, impaired motivation and concentration, and aggression; and reduced norepinephrine levels can also contribute to changes in sex, appetite, aggression, concentration, interest, and motivation (70). It seems that tumor-derived or tumor-initiated cytokines are able to dysregulate serotonin synthesis through different mechanisms. For instance, there are some tumors that can regulate the metabolism of 5-HT, increasing the local concentration of some metabolites or 5-HT itself (71). This has two main effects that have been postulated. On the one hand, the increase in 5-HT can stimulate the PI3K-Akt-mTOR pathway and therefore promotes tumor metabolism and thus tumor growth (72). On the other hand, this metabolic switch can drive a decrease in serotonin reserves in the brain, which is related to the appearance of depressive symptoms (71). In a similar manner, there are some tumors that may promote the activation of the enzyme indoleamine 2,3-dioxygenase (IDO), which converts tryptophan, a precursor of 5-HT, into kynurenine (KYN) (20). Importantly, these metabolic switches not only deplete serotonin synthesis but also have important neurotoxic effects on the brain (73). KYN is able to pass the blood–brain barrier (BBB) and is metabolized into quinolinic acid (QA), 3-hydroxykynurenine, and 3-hydroxyanthranilic acid by the microglia, exerting neurotoxic effects or into kynurenic acid (KA) in astrocytes, with neuroprotective actions (74). Patients with MDD appear to present reduced serum levels of KA, although there is no evidence of a decreased detection of this component in the brain, and the precise status of the KYN pathway in the brain must be fully studied (75). What seems clearer is that there is an imbalance between excitotoxic and neuro-protective agents in patients with MDD, and this fact can be also related to enhanced susceptibility to stress, inflammation, and gut dysbiosis (76).

However, other studies have also observed other correlations between cancer and monoamines. For instance, it seems that there is a correlation between dysregulation of the Ras oncogene family and impairment in serotonin and dopamine synthesis, resulting in the onset of MDD (77). Moreover, neuroinflammation and proinflammatory cytokines may impair dopamine synthesis, packaging, and release, correlating with anhedonia, fatigue, and psychomotor retardation (78). For instance, studies in non-human primates have demonstrated a pivotal modulatory role of interferon alpha (IFN-α), leading to a decreased expression of dopamine 2 receptors and reduced striatal dopamine release, possibly due to a conversion of the amino acid phenylalanine to tyrosine, resulting in depressive symptoms (5). Regarding norepinephrine, patients with depressive symptoms and feelings of low social support appeared to have higher tumor norepinephrine levels in ovarian cancer, which is directly correlated with tumor growth and stage (79, 80), thereby showing a biopsychosocial relationship between cancer and MDD. Similar to the previous monoamines, the role of some proinflammatory cytokines like TNF-α and IL-1 due to cancer or after cancer therapy appears to increase 5-HT and norepinephrine reuptake transporters, through activation of the p38 mitogen-activated protein kinase (MAPK), driving the onset of depressive symptoms (5).

Glutamate is another neurotransmitter also related to the development of MDD (81). An aberrant glutamate signaling is associated with neuronal excitotoxicity especially under inflammatory and abnormal glial conditions, as is the case of MDD (82). $X_{c}^{-}$ system is an antiporter that is also potentially involved in the release of glutamate to the extracellular space while importing cystine (83). This system is particularly important in cases of oxidative stress, as the entry of cysteine in the cell is critical in synthesizing the antioxidant glutathione (GSH) (20). Oxidative stress frequently co-occurs with neuroinflammation and cell stress, and it is also a major pathophysiological feature of MDD (84). Likewise, tumoral cells can also be a major source of glutamate, taking part in the development of cancer-induced depression (85). Thus, inhibiting the liberation of glutamate and targeting the $X_{c}^{-}$ system have been proposed as a promising anticancer approach *in vivo*. Other neurotransmitters such as GABA, with opposite effects to glutamate and acetylcholine, along with certain neuropeptides like neuropeptide Y, neurotensin, and oxytocin, can also play a pivotal role in the relationship between cancer and depression, having a tight relationship with chronic stress (86). The last one is oxytocin, which is altered in patients with MDD and is associated with different prodepressant and antidepressant mechanisms (87). Interestingly, this molecule has proven to exert important antitumoral effects in different types of cancer (88). However, further molecular studies are needed to unravel the biological basis of this link.

**Figure 1:**
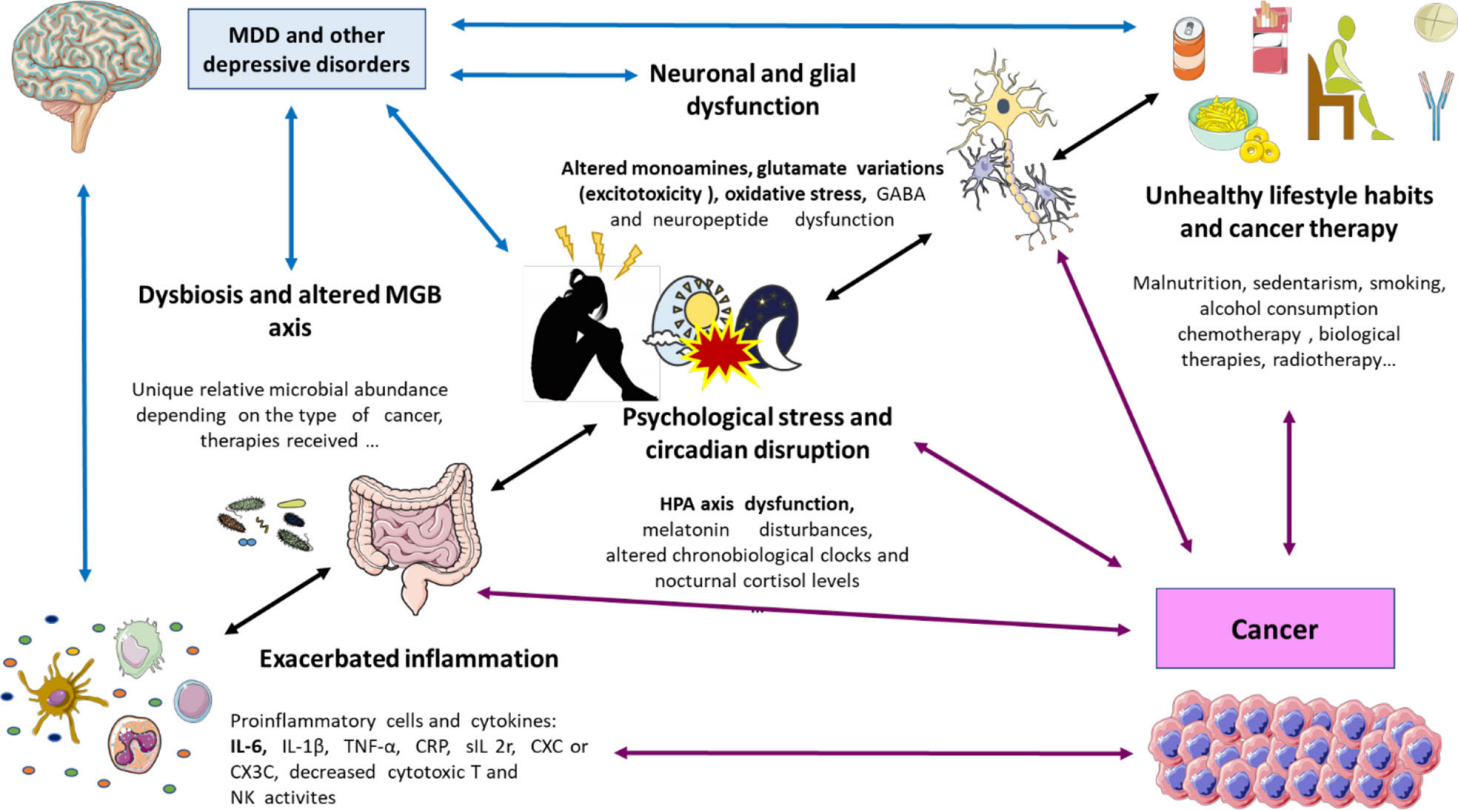
A global perspective on the biological links between cancer and depression. As shown, there are plenty of factors that are interrelated including an exacerbated inflammation (prominently due to augmented proinflammatory cells and cytokines like IL-6); dysbiosis (with different microbial profiles) and disrupted microbiota gut brain (MGB) axis; psychological stress and circadian disruption (prominently through HPA disruption, changes in melatonin, nocturnal cortisol, and circadian clocks); neuronal and glial dysfunction (changes in neuropeptides and neurotransmitters like monoamine metabolism, glutamate, and GABA); unhealthy lifestyle habits like smoking, malnutrition, and sedentarism; and the proper cancer therapy. IL, interleukin; MGB axis, microbiota–gut–brain axis; HPA axis, hypothalamic–pituitary–adrenal axis; GABA, gamma aminobutyric acid.

The multiple biological links explored in this section are shown in **Figure 1**.

## Clinical management of depression in oncological patients

### Diagnosis and depression assessment

Clinical diagnosis of depression is primarily based on the *Diagnostic and Statistical Manual of Mental Disorders*, 5th edition (DSM-V), which includes, at least during the period of 2 weeks, the presence of either depressed mood or anhedonia (referred to as the main criteria) and a minimum of four additional somatic or non-somatic symptoms like fatigue, suicidal ideation, weight loss or gain, feelings of worthlessness, and loss of attention (89). However, in patients with cancer, it is difficult to identify some symptoms necessary to assess the presence of depression, such as asthenia, weight loss, or insomnia, as these can be confused with symptoms caused by the tumor or by side effects of chemoimmunotherapy treatment (90). However, we cannot forget that the high workload of oncologists, as well as the difficulty in carrying out prospective studies in which adequate follow-up of the symptoms of depression vs. cancer is carried out, makes it even more difficult to establish adequate epidemiology of the current problem. In turn, patients may present MDD throughout their illness, from the initial study to palliative care, so they should undergo periodic reevaluations, something that is sometimes not available in daily clinical practice (91). Similarly, current evidence suggests that the prevalence of depression differs according to the type of tumor, being more representative in digestive, brain, genital tract, or hematological tumors (92). This leads to a generalized situation in which patients are underdiagnosed and are not being adequately treated from a psycho-oncological point of view. In addition, we must add that the spectrum of depressive disorders is very broad, which makes diagnosis even more difficult. For example, the patients may present mixed symptoms of anxiety/depression, minor depression, anxiety, etc. (93). However, to deal with this situation, standardized questionnaires have been implemented in recent years to diagnose depression in cancer patients. Among them, it must be highlighted the PHQ-15 or the GAD-7, although it is the Patient Health Questionnaire-4 (PHQ-4), in which anxiety and depression are evaluated with four short questions, offering the best results in routine clinical practice given the ease of its application (94–96). These questionnaires aid to establish which patients are eligible for further evaluation by a clinical psychologist or psychiatrist. Since these types of screening tools do not have possible adverse effects, they are beginning to be recommended on a day-to-day basis and allow for an adequate psycho-oncological approach to cancer patients (97). Therefore, patients should ideally be screened at the first oncology consultation, as well as at subsequent visits regardless of whether the patients are in remission, recurrence, or progression. This would aid to assess if their clinical manifestations can be treated with psychotropic drugs or different psychological approaches or if, oppositely, the symptoms are a consequence of tumor progression or the adverse effects of some therapeutic line (98). At the same time, we must emphasize that there are symptoms of depression and cancer that overlap, and we should suspect that patients present symptoms of depression when there is a lack of adherence to treatment, hopelessness, anger, rage, or other manifestations that are not consistent with the biopsychosocial situation of the patients (99). Therefore, the prevalence and diagnosis of depression in cancer patients are one of the current challenges in the multidisciplinary management of those affected by any type of malignant neoplasm and whose correct approach allows for improving the quality of life and prognosis of these patients.

### Medical care of depressed patients with cancer

In recent years, the advances conducted in the medical care of depression in patients with cancer have been translating into a substantial improvement in the quality of life of these patients and have become a quality standard in the different oncology units (7, 100). As mentioned earlier, the links between depression and cancer are multiple, including numerous metabolic, neuroendocrine, psychological, or immune pathways, without forgetting that depression is an adverse prognostic factor in itself (101, 102). This may be due to a decrease in adherence to chemotherapy treatments or an increase in suicide rates, among other possible causes (5). However, we cannot forget that it is difficult to analyze how median survival is affected by depression since numerous factors have to be taken into account, such as tumor stage, metastatic invasion, or the patients’ social and family support. Even so, the ultimate goal of early diagnosis of major depression in cancer patients is an improvement in the patients’ quality of life.

One of the main tasks of the doctor is to rule out secondary causes that are causing the depressive symptoms, such as hypercalcemia, hypothyroidism, or secondary effects of biological drugs such as interleukins or chemotherapy agents such as vinblastine or vincristine (103). With respect to the possible approach proposed for these patients, current evidence indicates that a combined therapy that integrates psychological interventions with pharmacological treatment is the most effective mechanism to deal with depression in cancer patients. Normally, this type of intervention is carried out by psycho-oncologists together with psychiatrists who carry out periodic evaluations of patients and assess modifications in their treatment. Numerous scales have also been designed, such as those previously mentioned (PHQ-15, GAD-7, or PHQ-4) in addition to the Beck Depression Inventory or the Hospital Anxiety and Depression Scale, which have been proven useful for depression screening and allow clinicians and psychologists to establish which patients are candidates for more intensive evaluations (104, 105). Regarding the use of pharmacotherapy, antidepressants have been shown to be superior to a placebo in MDD or depressive symptoms in cancer patients with few adverse effects (106). The drugs that are best tolerated with the greatest safety indexes are SSRIs. For example, its use has been studied together with the synchronous administration of chemotherapy without causing relevant adverse effects (107). Likewise, there are numerous drugs that can be used to treat these patients depending on the predominant symptomatology, such as atypical antidepressants (trazodone or mirtazapine) in the event that the main symptom is agitation or insomnia; psychostimulants such as methylphenidate improve attention or fatigue and weakness or benzodiazepines in the management of anxiety and insomnia (108–110). There is also a set of antidepressants that have been studied for their analgesic effect acting as adjuvants in pain management, such as duloxetine or desipramine, so they could be considered in cancer patients with chronic pain (111). However, antidepressants are not without risks since they are known to lower the seizure threshold and in the event that the patients present brain metastases, neoplasms of the central nervous system, or metastatic meningitis, neuroleptics (such as aripiprazole or other second-generation neuroleptics) in the management of depressive symptomatology, so antipsychotics are used in the treatment of depression in those fragile people with cancer who are susceptible more to the side effects (112). Despite this, some experts have indicated that a large proportion of cancer patients are not being optimally treated and are receiving insufficient doses, regimens that are not adapted to the patients’ predominant symptoms, or drugs that have fallen into disuse (113).

Another critical aspect in the multidisciplinary management of depression that is interesting in cancer is its prevention. This allows not only to anticipate the disease but also to deal more properly with this underdiagnosed pathology as well as to conduct a more exhaustive control of these patients in order to improve their quality of life. For this reason, in a meta-analysis carried out by Zahid et al., prophylactic doses of different drugs have been evaluated, as well as different psychotherapeutic interventions, obtaining promising results where the incidence of depression in oncological patients was lower compared to that in the control group that did not receive any of the previously described interventions (114). It should be noted that there are some pivotal limitations in the studies included in that meta-analysis, including a wide variety of possible biases observed, like allocation bias, randomization, or double-blinding. However, as we have previously commented, depression in cancer patients is very difficult to evaluate for different reasons, since the progression and therapeutic response to different chemoimmunotherapy lines generate a complex situation to be able to carry out clinical trials without biases, allowing relevant conclusions to be drawn. In this sense and as it will be subsequently discussed, a growing body of evidence is giving the lifestyle factor a pivotal role to prevent and aid in the clinical management of cancer and depression, as these factors appear to modulate a broad spectrum of biological and psychosocial factors implicated in the etiopathogenesis of both conditions.

## Lifestyle medicine as preventive, adjunctive, and global support in the management of cancer and depression

Some authors have noticed that the augmented incidence and prevalence of depression and cancer are importantly attributed to modernity, which appears as a consequence of a suboptimal lifestyle in which there is low physical activity, inadequate nutrition and rest, low exposure to sunlight, or psychological and social difficulties, among others (115, 116). Lifestyle medicine is a multidisciplinary field of knowledge defined by the American College of Lifestyle Medicine (ACLM) as “the use of evidence-based lifestyle therapeutic approaches, such as a predominately whole food, plant-based diet, physical activity, sleep, stress management, tobacco cessation, and other nondrug modalities, to prevent, treat, and, oftentimes, reverse the lifestyle-related chronic disease that’s all too prevalent” (117). In this sense, there is compelling evidence that lifestyle interventions are critical strategies for the prevention and management of patients with cancer and MDD (118–121).

In this section, we will review some of the most important lifestyle interventions in the field of cancer and MDD, with a focus on a) diet, b) physical activity, c) sleep, and d) psychological and social interventions. Despite not collecting this information in this section, it is of great relevance to focus on certain routines or lifestyle actions to avoid or limit, such as medication/substance abuse (including alcohol and smoking) or the misuse of modern technologies, which are crucial for achieving a healthy lifestyle (122).

### A) Dietary interventions

Diet could be considered a multitargeting pill, as it affects our organism at many different physiological and homeostatic levels. In the field of cancer and MDD, diet can modulate epigenetics, the HPA axis, inflammation, oxidative stress, metabolism, gut microbiota, neurotransmitter synthesis, and tryptophan-kynurenine metabolism, among other processes (123, 124).

Malnutrition (deficiency, excess, or imbalance of a wide range of nutrients) is one of the major contributors to a wide range of diseases, with detrimental consequences for the patients, affecting the function and recovery of every organ system (125). For instance, overweight and obesity, frequently related to malnutrition, appear to increase the risk to develop MDD, and in turn, this condition frequently leads to malnutrition, overweight, and obesity (126). Similarly, overweight and obesity contribute to increased mortality due to cancer (127), as well as an augmented risk of suffering from cancer, even in metabolically healthy overweight/obese adults (128). Despite nutritional deficiencies being rare in developed countries, suboptimal consumption of different micronutrients (vitamins and minerals) is frequent and also associated with an increased risk of cancer and MDD ( (129, 130). Moreover, there is a close correlation between malnutrition with psychological stress in cancer patients (131). Hospitalized oncological patients appear to be frequently malnourished, and recent studies have found that these patients are 6.29 times more likely to present depressive symptomatology in comparison to those who are well-nourished (132). Worsening of the nutritional status in patients with advanced stages of cancer appears to exacerbate depressive symptoms (133).

In this context, nutritional intervention can be of great aid in the context of MDD and cancer. There are different potential strategies for this goal. There are nutrients with notable relevance to many physiological processes, with a potential preventive and adjunctive therapeutic action. These are the cases of nutraceuticals, which can be defined as food ingredients and dietary supplements, although there is some controversy regarding their nomenclature (134, 135). Moreover, there are a group of foods with interesting nutritional value that may contain multiple nutraceuticals and exert synergic effects. Unlike dietary supplements, foods contain their own matrix, which can be responsible for these combined benefits (136). In the case of oncological patients, nutritional intervention has proven moderate or limited evidence, despite the nutritional status being associated with poorer survival, decreased treatment completion, and higher healthcare consumption (137, 138). These results can be due to the heterogeneity of available studies and the need for personalized nutrition when considering nutritional intervention. However, the evidence seems to indicate that a healthy dietary pattern diminishes the risk of suffering from colorectal and breast cancers, especially in postmenopausal, hormone receptor-negative women (139). Moreover, high adherence to healthy dietary patterns like the Mediterranean diet (MedDiet) appears to be related to lower odds of MDD and lower risk of cancer mortality in the general population, and all-cause mortality among cancer survivors as well as respiratory, colorectal, gastric, liver, bladder, and head and neck cancer risks (140, 141). Regarding the role of diet as adjunctive therapy, few studies have been conducted. An inverse relationship was observed between a healthy diet, coffee, fish, or dietary zinc and the onset of MDD, and probiotics, omega 3 polyunsaturated fatty acids, and acetyl-carnitine supplementations show moderate-quality evidence for depression treatment (142). In the case of cancer patients and cancer survivors, there is little evidence supporting the use of a specific group of foods to improve the clinical outcome and prevention of recurrence, although high adherence to healthy dietary patterns like MedDiet appears to exert potential benefits in this sense (143, 144). There is insufficient evidence to support the efficacy of a low carbohydrate ketogenic diet, whose efficacy can only be extrapolated in some types of cancer in animal models (145, 146). However, the high consumption of vegetables and fruits, due to their high content of polyphenols, can be a potential strategy for further studies in cancer and depression (147, 148).

### B) Encouraging physical activity

In a similar manner to diet, physical activity (PA) also has multiple targets, influencing the proper tumor biology and the brain through the modulation of oxidative stress, inflammation, overweight and obesity, neurotransmission, neurogenesis, cognitive processes, and memory due to the secretion of certain products like endorphins, myokines, and enhancing thermogenesis (149, 150). Conversely, sedentarism is one of the greatest contributors to the development of multiple chronic maladies, including MDD and cancer (151). Indeed, some authors have found a direct correlation between sedentary time with higher scores of depressive symptoms (152). Because of that, the prescription of PA is actually considered a potential preventive and adjunctive support for the management of MDD in patients with cancer.

Different studies have shown how physical activity decreases sarcopenia by improving muscle strength, quality of life, and chronic fatigue in cancer patients (153). Current expert recommendations such as those reported by the American College of Sports Medicine (ACSM) claimed that aerobic training performed three times per week and for at least 12 weeks or twice weekly with combined aerobic plus resistance training can significantly reduce depressive symptoms in cancer survivors during and after treatment, although there are some controversial data regarding the most adequate dose of exercise for depressive patients with cancer (154). A meta-analysis conducted by Brown et al. analyzed the benefits of up to 40 different types of physical exercises (aerobic, relaxation, and muscle hypertrophy) in approximately 3,000 patients with hematological neoplasms, colon, breast, or prostate cancer and from 37 different studies. Interestingly, they obtained a slight improvement in depressive symptomatology in patients with different malignant neoplasms, with a dose-response increase fashion following aerobic exercise. Resistance training alone did not seem to present benefits for these patients, such as those that stimulate the body’s physical endurance capacity in the face of sustained effort, through both aerobic or anaerobic efforts, as well as local (focused) or general (whole body) efforts. Moreover, the maximum benefits were obtained in breast cancer survivors aged between 47 and 62 years and when exercise was supervised by a professional (155). Consistently with these data, Craft et al. (156) found modest positive outcomes from PA to improve depressive symptoms in oncological patients, with the largest benefits when the programs were supervised or partially supervised, not conducted at home, and at least 30 min in duration per session. Another meta-analysis conducted by Patsou et al. (157) concluded that when progressive exercise programs are adapted by cancer survivors according to their individual needs, capabilities, and preferences, they offer a valid alternative to depression mood management. Cancer treatment, especially chemotherapy, appears to have negative effects at various cognitive levels in cancer patients, leading to the onset of depressive symptoms that could be ameliorated by moderate and vigorous levels of PA (158). Conversely, for breast cancer survivors, light-to-moderate but not vigorous PA levels exert the maximum antidepressant benefits (159).

The earlier PA is adopted after a cancer diagnosis, the more benefits it will bring. In this sense, Salam et al. (160) have noticed that statistically significant improvements in levels of depression were identified following the exercise intervention, supporting that post-diagnosis physical activity leads to a decrease in depression scores. In more detail, post-diagnosis exercise as a part of the daily routine led to a 37% reduction in the rate of breast cancer-specific mortality and a decrease of 39% in the all-cause mortality rate. However, it is important to underline that when depressive symptoms present, PA can be experienced as more difficult and demanding, eventually leading to patients engaging in less exercise (161). Thus, other strategies should be considered in conjunction with PA. For instance, group training can be an interesting approach for some patients with cancer, as they may achieve the benefits not only from physical activity but also from social interactions and support (150).

Overall, despite a growing body of evidence supporting the imperative need for PA as an adjunctive and potential lifestyle intervention in patients with cancer and depression, some questions remain to be answered yet. Further studies providing more high-quality evidence for the efficacy of PA in depressed patients with cancer are needed, as well as additional efforts examining the better dose of exercise in this vulnerable population (154).

### C) Sleep-based interventions

As explained before, altered circadian rhythms are a major feature of patients with MDD and cancer. In turn, sleep disturbances are importantly related to depression persistence and cancer progression in these patients, thereby representing an important pathophysiological link between both conditions (162). Thus, circadian and sleep-based interventions have offered promising results regarding the prevention and management of both cancer and depression.

In this context, different strategies can be considered here, including pharmacological and non-pharmacological approaches. Melatonin supplementation is the pharmacological intervention most widely studied. Despite some studies that have obtained some positive results from its prophylactic use in the field of MDD and alleviation of depressive symptoms, the effectiveness of this substance appears to be limited (163, 164). In patients with cancer, an intervention with 6 mg of oral melatonin or placebo for 3 months in women with breast cancer after surgery showed that melatonin significantly reduced the risk of depressive symptoms (38). Similarly, 20 mg of melatonin before and during the first cycle of adjuvant chemotherapy for breast cancer exerted neuroprotective actions for these women, improving sleep quality, depressive symptoms, and cognitive functions (165).

However, non-pharmacological interventions are perhaps more adequate strategies to influence sleep and circadian rhythms. A meta-analysis conducted by Gee et al. (166), with a sample of 5,908 patients and 49 clinical trials, concluded that non-pharmacological sleep interventions are effective in reducing the severity of depression, particularly in clinical populations. Different types of non-pharmacological sleep interventions have been studied, including a) relaxation training, b) sleep restriction, c) stimulus control therapy, d) cognitive behavioral therapy (CBT), and e) psychoeducation/sleep hygiene rules (167, 168). Of them, there have been some studies demonstrating the benefits of CBT in the amelioration of depressive symptoms in cancer patients, also improving their overall quality of life (169). In the case of breast cancer patients, the maximum benefits of CBT were observed in younger patients and those suffering from more severe insomnia (170). Similar results were observed in cancer survivors, and importantly, the benefits of CBT were also found even 3 months after the completion of this therapeutic approach (171). Improving sleep hygiene can also have the potential to reduce depressive symptoms in cancer patients, as there is a direct correlation between poor sleep hygiene with fatigue, sleepiness, anxiety, depressive symptoms, and worse insomnia in men with prostate cancer (172). In this sense, light exposure and, especially, sunlight exposure, are a prominent and potential approach for improving sleep quality and ameliorating depression in cancer patients (173, 174). These benefits can be maximized if combined with PA outdoors, including different intensities like light, moderate, and vigorous exercises.

### D) Psychosocial interventions

As previously discussed, psychological stress, feelings of loneliness, and social isolation are critical drivers of MDD in patients with cancer, entailing multiple biological mechanisms especially mediated by an altered HPA axis. Because of that, psychosocial interventions are great approaches to preventing and aiding in the clinical management of oncological patients with depression (175). A meta-analysis including 78 studies reported that psychological intervention is of great aid to improve the overall quality of life in patients with cancer, especially when combined with the management of depressive symptoms (176). Patients with mild to moderately severe depression may benefit the most from the combination of antidepressants and psychotherapy, although for patients with severe depression, psychotherapy appears to move into the background (177). In the case of patients with advanced cancer, psychotherapy can be especially useful for patients with depressive symptoms, but not with MDD (178). However, psychological intervention with different approaches such as CBT, mindfulness, and group support therapy has proven to be useful in causing an improvement in depressive symptoms in cancer patients and cancer survivors (179–181). It is also true that there are numerous biases in this type of intervention since it is difficult to analyze the type of intervention that is best suited to each patient or the improvement in quality of life and depressive symptoms in patients whose staging, prognosis, or oncological treatment varies among them (182). In this sense, some studies have reported better results of this therapy when performed individually instead of in groups (179). The integration of pharmacotherapeutic treatment with the psycho-oncological approach has been shown in numerous clinical trials to cause an improvement in the quality of life of these patients. However, there are numerous difficulties in the development of clinical intervention trials that evaluate the effect of the use of different antidepressants and psychotherapy. For example, difficulties have been reported in recruiting patients with cancer and depression in these studies, as well as the refusal by patients or physicians to use a placebo in trials or to establish appropriate control groups in the context of psychotherapy, leading to strong side effects. Placebo in the same (183). In the same way, the approach of the patients’ relatives and caregivers should take on important importance in order to analyze a possible intrafamily burnout syndrome and prevent the appearance of anxiety, depression, or anticipated pathological mourning. Family overload has been studied, and an increased incidence of depression has been observed in male family members of women who have breast cancer (184). The entire psycho-oncological approach not only improves the quality of life of cancer patients but also improves adherence to treatment, improves follow-up after active treatment, and ultimately improves the survival of cancer patients.

However, authors such as Komatsu et al. have described the benefits of implementing self-help recommendations in patients with breast cancer in order to prevent depression and anxiety and improve quality of life, although there are no recommendations in the form of books or pamphlets that have managed to reduce depression in patients undergoing chemotherapy (185). We must emphasize that the preventive management of depression is complex despite the numerous psychopharmacological approaches that have been studied. We have previously indicated how the multifactorial etiopathogenesis of depression makes not only the diagnosis and treatment of this entity difficult, but also prevention remains complex, and numerous studies must be carried out in order to analyze different preventive interventions in cancer patients to improve the quality of life and reduce the incidence of depression and anxiety associated with this entity. In this sense, numerous studies have shown how psychopharmacological drugs in cancer can have effects on cancer patients at different biological levels and can be another tool for the comprehensive management of these patients (186–188).

## Conclusions

**Figure 2:**
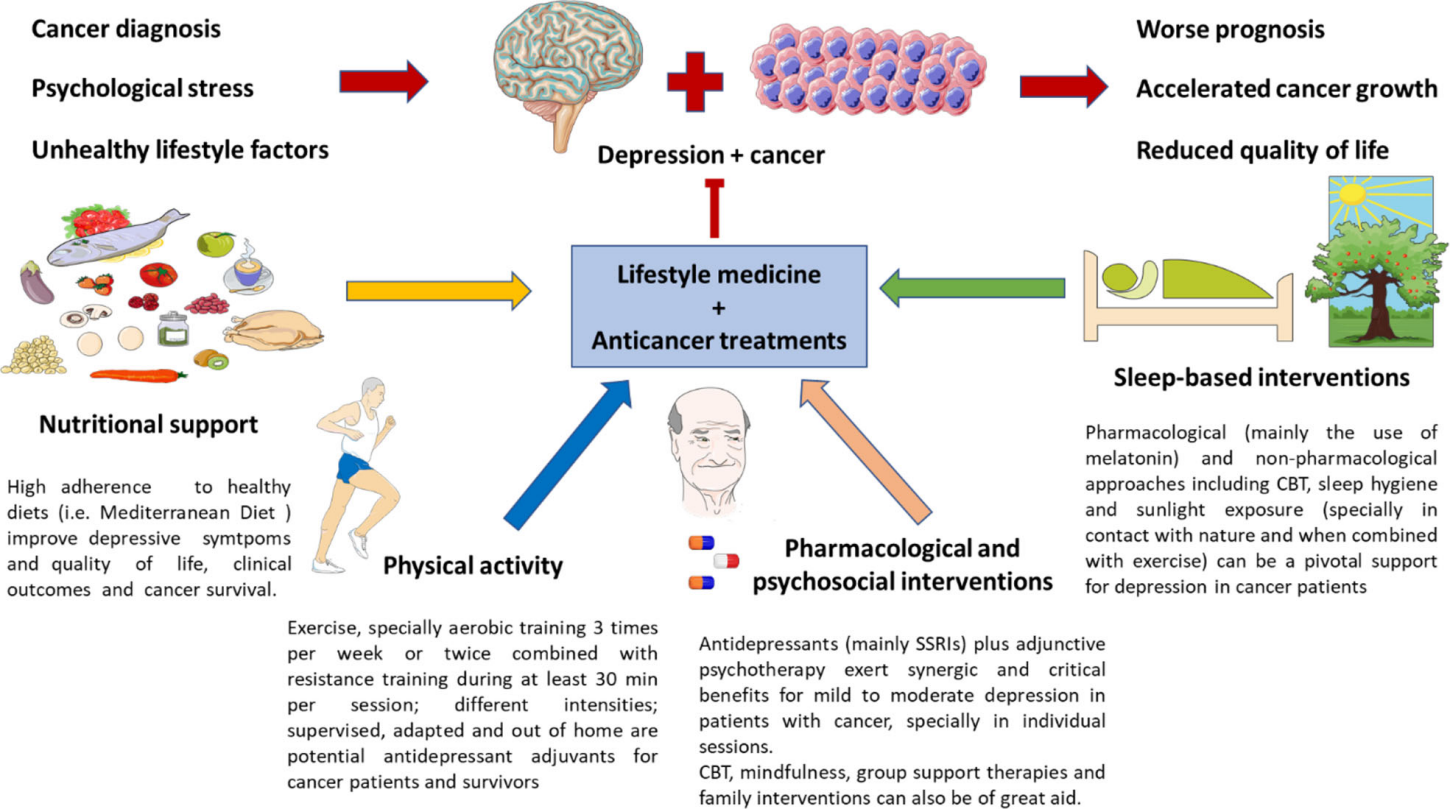
Lifestyle medicine in the clinical management of depression in patients with cancer. As previously mentioned, due to the proper diagnosis of cancer and its emotional impact, psychological stress, and unhealthy habits, patients with cancer can suffer from depressive disorders, which are related to a poorer prognosis, accelerated cancer growth, and a reduced quality of life. Nutritional support, together with encouraging physical activity, psychosocial plus pharmacological adjuvants and sleep-based interventions are critical for preventing and ameliorating depressive symptoms in cancer patients. However, it cannot be denied that some of these measures are not easy to implement in this population. Thus, the main evidence collected up to date in each field is briefly exposed in this picture.

In this work, we intended to explore the complex and multiple links between two single and quite heterogeneous entities like cancer and MDD. Despite their singularities, they share many pathophysiological mechanisms like an altered HPA axis, circadian disruption, inflammation, gut dysbiosis, and changes in the endocrine and nervous systems, which should be understood as interconnected factors encompassed in the PNIE. Patients with MDD have an increased risk of suffering from cancer, and patients with cancer frequently exhibit depressive symptoms due to the emotional impact of the disease. Moreover, the co-occurrence of cancer and MDD is associated with a worse prognosis for the patients, accelerated cancer development, reduced quality of life, and survival. Thus, the diagnosis, prevention, and therapy of depression in patients with cancer are essential for improving the clinical management of these patients, although it is an issue undoubtedly complex. Notwithstanding some improvements have been done in this area, further translational approaches are required in this population. Lifestyle medicine and implementing healthy lifestyle habits are critical strategies to prevent or diminish the risk of suffering from both MDD and cancer. Moreover, they represent imperative adjunctive support to limit the impact of cancer and depression, boosting the therapy received and limiting its side effects, therefore improving the quality of life of these patients. The most relevant evidence collected about this topic is summarized in **Figure 2**. Overall, despite there being multiple difficulties in this field and no single formula, it must be individualized and adapted to the patients; the cumulative evidence encourages the numerous benefits of implementing this kind of approach in healthcare systems for depression in patients with cancer.

## Author contributions

All authors contributed to the article and approved the submitted version.

## Funding

The study was supported by the Comunidad de Madrid (B2017/BMD-3804 MITIC-CM) and HALEKULANI, S.L. The funder was not involved in the study design, collection, analysis, interpretation of data, the writing of this article or the decision to submit it for publication.

## Conflict of interest

The authors declare that the research was conducted in the absence of any commercial or financial relationships that could be construed as a potential conflict of interest.

## Publisher’s note

All claims expressed in this article are solely those of the authors and do not necessarily represent those of their affiliated organizations, or those of the publisher, the editors and the reviewers. Any product that may be evaluated in this article, or claim that may be made by its manufacturer, is not guaranteed or endorsed by the publisher.
